# A novel Laennec's capsule tunnel approach for pure laparoscopic left hemihepatectomy: a propensity score matching study

**DOI:** 10.3389/fsurg.2023.1136908

**Published:** 2023-05-25

**Authors:** Jianlin Lai, Junyi Wu, Yannan Bai, Yifeng Tian, Yaodong Wang, Funan Qiu

**Affiliations:** ^1^ Shengli Clinical Medical College of Fujian Medical University, Fuzhou, China; ^2^ Department of Hepatobiliary Pancreatic Surgery, Fujian Provincial Hospital, Fuzhou, China

**Keywords:** novel surgery, Laennec's capsule, extrahepatic Glissonian approach, laparoscopic left hemihepatectomy, propensity score matching

## Abstract

**Background:**

With the development of laparoscopic hepatectomy, there are different surgical approaches and pedicle anatomical methods for laparoscopic left hepatectomy. Combined with our practical experience, we proposed a method of transhepatic Laennec membrane tunnel for laparoscopic left hemihepatectomy (LT-LLH) and investigated the feasibility by comparison with the extrahepatic Glissonian approach for laparoscopic left hemihepatectomy (GA-LLH).

**Patients and methods:**

The data of patients who underwent laparoscopic left hepatectomy in the Department of Hepatobiliary Pancreatic surgery of Fujian Provincial Hospital from December 2019 to March 2022 were analyzed retrospectively. Among them, 45 cases underwent laparoscopic left hemihepatectomy with an extrahepatic Glissonian approach, and 38 cases underwent laparoscopic left hemihepatectomy via transhepatic Laennec membrane tunnel approach. A 1:1 propensity score matching (PSM) method was performed to compare the perioperative indexes and long-term tumor prognosis between the two groups.

**Results:**

After 1:1 PSM, 33 patients in each group were selected for further analysis. Compared with the GA-LLH group, the operation time of the LT-LLH group was shorter. There was no significant difference in the incidence of total complications between the two groups. Moreover, no statistical differences were found in disease-free survival and overall survival between the two groups.

**Conclusion:**

It is safe, faster, and convenient for selective appropriate cases to carry out laparoscopic left hemihepatectomy through the hepatic Laennec membrane tunnel, which is suitable for clinical promotion.

## Introduction

With the development of laparoscopic technology and instruments, laparoscopic hepatectomy has almost no restricted area, and surgical techniques are more mature (1). However, Laparoscopic left hemihepatectomy was performed safely with efficacy in specialized centers and was considered the most acceptable and appropriate candidate for a standard of care as early as 2012 (2), the technique of this operation was still worth optimizing and exploring. In recent years, many scholars have put forward their own experiences and skills on the approach of liver parenchyma and the anatomical method of left hepatic pedicle in laparoscopic left hemihepatectomy, which has played a positive role in the development of laparoscopic hepatectomy.

Different hepatic parenchyma approaches include the caudal ventral approach, caudal dorsal approach, dorsal cephalic approach, etc. (3–7), which have different characteristics and unique advantages, and the most commonly used and classic approach is the caudal ventral approach (7). For the management method of the hepatic pedicle, the earliest is the conventional hilar approach, which gradually develops into the extrahepatic Glissonean approach, and then the Glissonean approach can be accomplished intrahepatically and extrahepatically (8). The extrahepatic Glissonean approach is considered to be safe and feasible with shorter operation time, less bleeding, and better perioperative prognosis than the conventional hilar approach (9). In 1802, R.T.H. Laennec first described the thin fibrous membrane as a structure different from the serous membrane and named it Laennec's capsule (10). In 2017, Sugioka et al. presented systematic extrahepatic Glissonean isolation based on Laennec's capsule. The concept of Laennec's capsule was re-proposed and the concept of four anatomical landmarks and six “Gates” (11) could be used for liver dissociation, hepatic pedicle separation, hepatic vein separation, and anatomical hepatectomy (12–14).

Therefore, we combine the caudal ventral approach of the hepatic parenchyma, Laennec's capsule anatomy theory, and Glissonean approach to summarize a new type of laparoscopic left hepatectomy, which is called Laennec membrane tunnel laparoscopic left hepatectomy (LT-LLH). As far as we know, the above procedures have not been summarized at present. Therefore, the purpose of this study is to provide our preliminary experience in LT-LLH and to compare the surgical outcomes with the conventional Glissonean approach based on PSM analysis.

## Patients and methods

### Patient selection

Between December 2019 and March 2022 the patients who underwent laparoscopic left hepatectomy in the Department of Hepatobiliary Pancreatic surgery of Fujian Provincial Hospital were analyzed retrospectively. The inclusion criteria were: (1) male or female aged from 18 to 75 years old, (2) laparoscopic left hemihepatectomy with extrahepatic Glissonean approach, (3) hepatocellular carcinoma or hepatolithiasis, and (4) liver function classified as Child–Pugh class A or B. The exclusion criteria were the following: (1) laparoscopic left hemihepatectomy requiring lymph node dissection or combined with caudate lobectomy; (2) giant liver cancer (>10 cm) with limited space; (3) the right anterior hepatic bile duct originates from the left hepatic duct or other variations of blood vessels and bile ducts; (4) extrathecal dissection cannot be performed due to left hepatic duct stones; (5) the Laennec's capsule cannot be dissected due to inflammation or tumor. All patients' therapeutic regimen and all operations were performed by the same surgical team at the same period.

According to the different surgical methods, the patients were divided into Laennec membrane-tunnel approach group (LT-LLH) and conventional Glissonean anatomical approach (GA-LLH) group. In addition, liver volume, hepatic vessels, and bile ducts were assessed by three-dimensional reconstruction in all patients preoperatively. Informed consents were obtained in accordance to the Helsinki Declaration and written informed consent was obtained from each participant in the study. The study was approved by the Ethics Committee of Fujian Provincial Hospital.

### Surgical procedure

The operation was performed under general anesthesia by endotracheal intubation. The patient took the supine position with split legs and the chief surgeon stood on the right side of the patient. The laparoscopic operation holes were performed by the 5-hole method, and the trocar sheaths were fan-shaped around the left liver. Carbon dioxide pneumoperitoneum was established to maintain intra-abdominal pressure of 12–14 mmHg.

**Figure 1:**
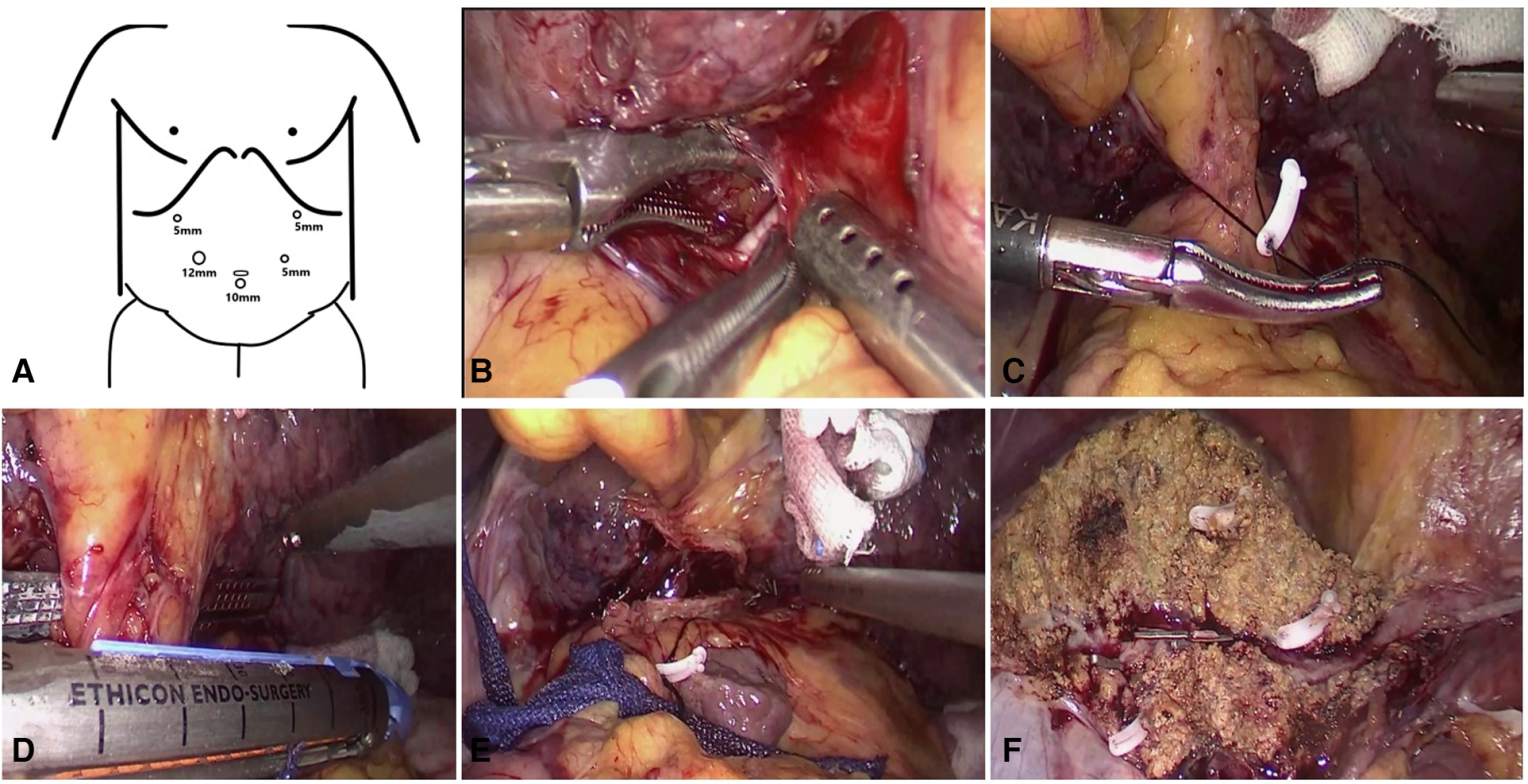
Some key surgical techniques of the extrahepatic Glissonian approach for laparoscopic left hemihepatectomy. (**A**) The trocar location of laparoscopic left hemihepatectomy. (**B**) Ultrasonic scalpel opened the hepatic hilar plate, separated the left hepatic pedicle at the boundary of the left-right Glisson sheath, then penetrated the tunnel behind the left hepatic pedicle. (**C**) Completely separated and encircled the left hepatic pedicle and placed silk thread or cuff to pull the left hepatic pedicle. (**D**) The laparoscopic linear stapler cut off the left hepatic pedicle. (**E**) The section of the left hepatic pedicle after disconnection. (**F**) Liver plane after left hemihepatectomy.

Conventional Glissonean anatomical approach group: Ultrasonic scalpel opened the hepatic hilar plate, separated the left hepatic pedicle at the boundary of the left-right Glissonean sheath or the right side of the left portal vein, opened part of the hepatic parenchyma if necessary, penetrated the tunnel behind the left hepatic pedicle, completely separated and encircled the left hepatic pedicle and placed silk thread or cuff to pull the left hepatic pedicle, and cut off the left hepatic pedicle with the laparoscopic linear stapler (Figure 1) after confirming the blood flow into the liver by endoscopic ultrasound.

**Figure 2:**
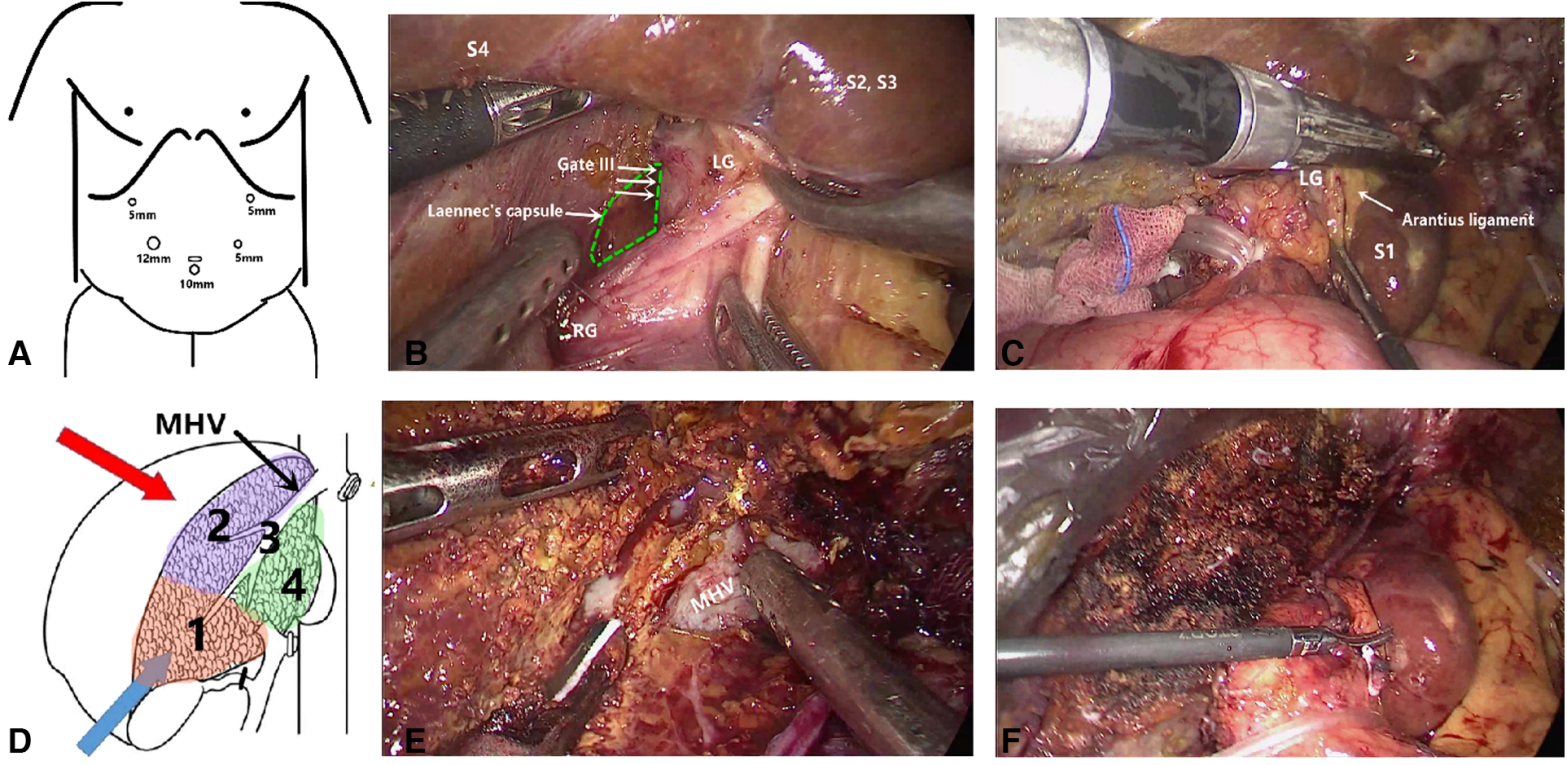
Some key surgical techniques of the transhepatic laennec membrane tunnel for laparoscopic left hemihepatectomy. (**A**) The trocar location of laparoscopic left hemihepatectomy. (**B**) The Gate III was opened with an ultrasonic knife at the left side of the right anterior Gleason's sheath and then a tunnel was established with an aspirator. (**C**) The left hepatic pedicle was severed on the ventral side of arantius tube with laparoscopic linear stapler. (**D**) The sequence of liver parenchyma dissection was shown. (**E**) Hepatic parenchymal dissection was performed along the middle hepatic vein. (**F**) Liver plane after left hemihepatectomy. Gate III: the right edge of the Glissonean pedicle root of the umbilical portion.

Laennec membrane-tunnel approach group (Supplementary Video): When blocking the first hepatic hilum, the membrane tissue between the liver parenchyma and the hepatic hilar plate at the left and right hepatic pedicle bifurcation was opened with an ultrasonic scalpel on the left side of the right anterior sectional Glissonean pedicle (the left edge of the bile duct of the right anterior lobe), that is Gate III (the right edge of the Glissonean pedicle root of the umbilical portion), and then a tunnel was established with aspirator. The left hepatic pedicle can be severed once a tunnel was built through Laennec membrane. Through the tunnel, the left and right hepatic ischemic lines were obtained after clamping the left hepatic pedicle with Endo-GIA. Compared with Glissonean anatomical approach group, it didn't need to completely separated and encircled the left hepatic pedicle. After the left hepatic pedicle was reconfirmed by endoscopic ultrasound during operation, the left hepatic pedicle was severed on the ventral side of arantius tube with laparoscopic linear stapler (Figure 2).

### Surgical outcomes and postoperative management

The two groups of patients received the same postoperative care by the same group of surgeons. Blood routine and liver function tests were performed on the 1st, 3rd, 5th and 7th day after operation. All patients were followed up with a standardized follow-up program. For the malignant tumors, liver-enhanced CT scans or liver MRI examinations were performed 1 month after surgery, then every 3 months for 2 years, and every 6 months after 2 years. Postoperative recurrence was defined as the appearance of new lesions with HCC features on follow-up CT or MRI.

The perioperative analyzed variables were included operation time, blood loss, intraoperative blood transfusion, conversion rate, postoperative hospital stay, postoperative liver function, postoperative complications (according to Clavien-Dindo grade) and mortality.

### Statistical analysis

The PSM analysis is a useful method that is widely used in retrospective studies to reduce confounding and selection bias. In our research, the LT-LLH group and GA-LLH group were compared with a 1:1 PSM analysis in an attempt to minimize intergroup disparities. A propensity score for each patient was calculated by logistic regression according to the imbalanced variables, and a 1:1 nearest-neighbor matching method was performed between the two groups. The variables involved in the PSM analysis include age, BMI, ASA grade, liver cirrhosis, previous dominant surgery, comorbidities, Largest tumor size, etc.

The patient characteristics were expressed as mean ± standard deviation or median of interquartile range for continuous variables, frequencies and proportions for categorical variables. Continuous data were analyzed by *t*-test or Mann-Whitney-Wilcoxon test, and categorical variables were analyzed by chi-square test or Fisher's exact test. Survival curves were estimated by the Kaplan–Meier method with log-rank comparison. *P* values less than 0.05 were considered statistically significant. Statistical analyses and PSM were performed using SPSS version 22.0.

## Results

### Baseline characteristics

A total of 83 patients who met the study criteria were enrolled in this study, of which 45 patients were treated with GA-LLH and 38 patients with LT-LLH. The baseline characteristics of all were summarized in Table 1. The two groups differed before PSM in term of age (*P* = 0.043), complication (*P* = 0.039) and tumor diameter (*P* = 0.007). After PSM, 33 patients in each group matched well, and baseline demographic data were comparable.

**Table 1:** Patient characteristics before and after propensity score matching.

	Before PSM			After PSM		
	GA-LLH group	LT-LLH group	*P* value	GA-LLH group	LT-LLH group	*P* value
	*n* = 45	*n* = 38		*n* = 33	*n* = 33	
Age, years	61.61 ± 9.40	61.52 ± 7.79	0.043	60.89 ± 10.37	60.76 ± 11.25	0.053
Sex, (M/F)	30:15	28:10	0.482	22:11	23:10	0.791
BMI, kg/m^2^	23.69 ± 2.8	22.78 ± 3.0	0.157	22.34 ± 2.9	22.45 ± 3.1	0.882
HBV carrier	15 (33.3%)	12 (31.6%)	0.314	12 (36.4%)	10 (30.3%)	0.602
Liver cirrhosis	22 (48.9%)	12 (31.6)	0.131	15 (45.5%)	10 (30.3%)	0.205
ASA grade			0.713			0.802
I	29 (64.4%)	23 (60.5%)		20 (60.6%)	19 (57.6%)	
II	16 (35.6%)	15 (39.5%)		13 (39.4%)	14 (42.4%)	
Previous abdominal surgery	10 (22.2%)	3 (7.9%)	0.074	5 (15.2%)	2 (6.0%)	0.230
Comorbidities	12 (28.9%)	4 (10.5%)	0.039	8 (24.2%)	4 (12.1%)	0.202
Cholangitis	5	4	0.932	5	4	0.720
Choledocholithiasis	8	5	0.564	7	5	0.523
Preoperative AFP			0.757			0.741
Increased (≥400 ng/ml)	7 (15.6%)	5 (13.2%)		6 (18.2%)	5 (15.2%)	
Not increased (<400 ng/ml)	38 (84.4%)	33 (86.8%)		27 (81.8%)	28 (84.8%)	
TB, µmol/L	15.62 ± 6.3	14.32 ± 5.2	0.314	16.21 ± 7.1	15.45 ± 5.8	0.636
ALB, g/L	41.85 ± 3.5	42.34 ± 2.8	0.489	42.20 ± 3.8	42.87 ± 4.1	0.494
AST, IU/L	42.68 ± 15.6	48.21 ± 17.6	0.133	43.28 ± 16.7	44.51 ± 18.3	0.776
ALT, IU/L	53.22 ± 20.5	52.34 ± 25.3	0.862	52.96 ± 22.7	54.21 ± 28.3	0.844
Largest tumor size, cm	6.5 ± 2.5	4.5 ± 4.0	0.007	5.2 ± 2.9	4.3 ± 4.0	0.299

### Operation and postoperative outcomes

Table 2 compared the perioperative outcomes of both groups after PSM. Compared with LT-LLH group, the operation time of GA-LLH group was significantly longer. The amount of blood loss in the LT-LLH group was similar with the GA-LLH group (*P* = 0.130). In the GA-LLH group, 3 cases were converted to laparotomy because of intraoperative bleeding, intra-abdominal adhesion and stone incarceration. In the LT-LLH group, 2 cases were converted to laparotomy because of abdominal adhesion and stone incarceration. There was no significant difference in postoperative outcome, recovery of liver function, postoperative hospital stay, ALT, AST, TB levels and total complications between the two groups. Similarly, there was no significant difference in the type of complications and the incidence of complications of grade II or above between the two groups. During the study, there were no death in both groups.

**Table 2:** Surgical characteristics and surgical outcomes after propensity score matching.

	GA-LLH group	LT-LLH group	*P* value
	*n* = 33	*n* = 33	
Laparoscopic left hemihepatectomy with CBDE	8 (24.2%)	6 (18.2%)	0.547
Blood loss (ml)	210 ± 120	185 ± 100	0.130
Blood transfusion, *n* (%)	5 (15.2%)	3 (9.1%)	0.709
Operation time (minutes)	220 ± 65	180 ± 80	0.029
Pringle maneuver, *n* (%)	30 (90.1%)	33 (100%)	0.076
Conversion to open laparotomy, *n* (%)	3 (9.1%)	2 (6.1%)	0.642
Postoperative hospital stay (days)	8.6 ± 3.5	7.5 ± 4.5	0.2718
Mortality, *n* (%)	0	0	-
Overall complications, *n* (%)	6 (18.2%)	5 (15.2%)	0.741
Clavien-Dindo grade, *n* (%)			
I	4 (12.1%)	3 (9.1%)	0.752
II	2 (6.1%)	2 (6.1%)	1.000
III	0	0	-
Clavien-Dindo grade II and above, *n* (%)	2 (6.1%)	2 (6.1%)	1.000
Postoperative liver function			
POD 1			
TB, µmol/L	20.34 ± 7.2	19.76 ± 8.0	0.758
AST, IU/L	155.89 ± 45.3	168.44 ± 50.2	0.290
ALT, IU/L	178.56 ± 55.9	188.62 ± 66.3	0.508
POD 3			
TB, µmol/L	25.45 ± 5.3	24.33 ± 6.3	0.437
AST, IU/L	105.35 ± 56.4	113.29 ± 48.2	0.541
ALT, IU/L	128.87 ± 66.3	135.34 ± 55.7	0.669
POD 5			
TB, µmol/L	17.56 ± 7.7	18.43 ± 6.9	0.631
AST, IU/L	30.46 ± 10.8	28.87 ± 8.9	0.516
ALT, IU/L	45.39 ± 8.7	42.32 ± 8.2	0.145

### Long-term oncological outcomes

**Figure 3:**
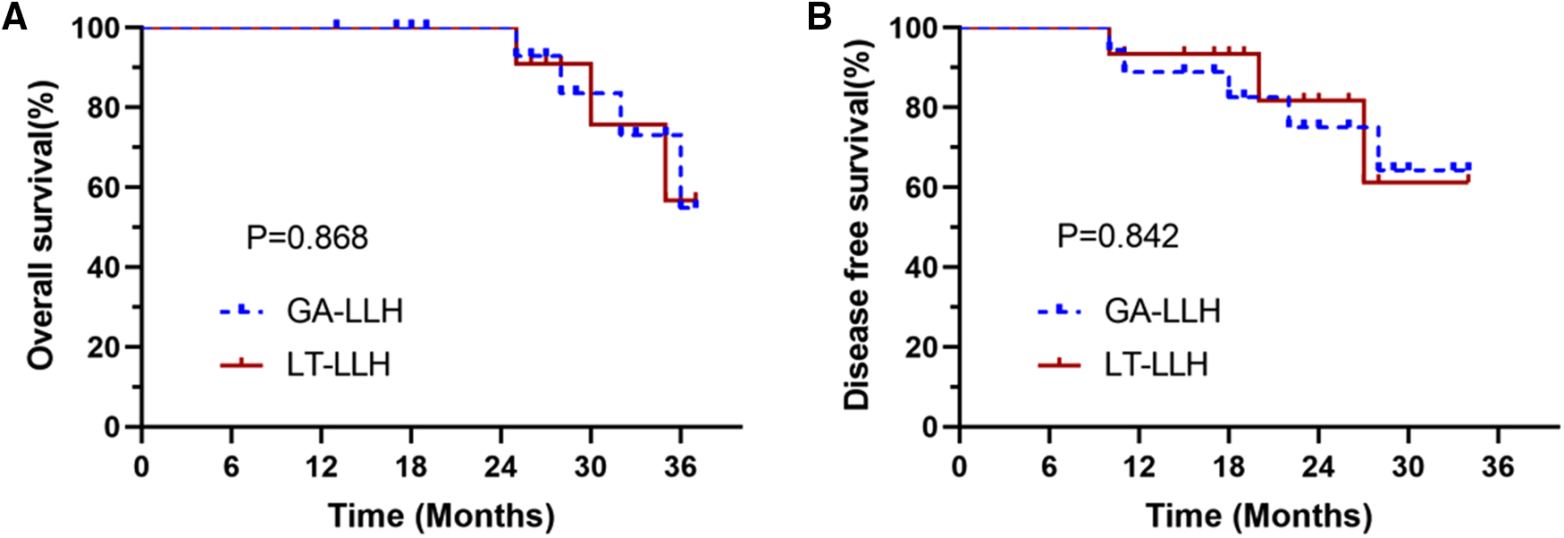
The survival curve between the conventional glissonean anatomical approach and the laennec membrane-tunnel approach: (**A**) overall survival (OS) rates and (**B**) disease-free survival (DFS) rates.

Patients with HCC were followed up to March 2022 and the long-term oncology results of the two groups were compared. The disease free survival (DFS) and overall survival (OS) curves are shown in Figure 3. The median follow-up period of LT-LLH group and GA-LLH group was 19.5 and 20.6 months respectively. There was no significant difference between DFS (*P* = 0.842) and OS (*P* = 0.868). The 1- and 3-year OS rates were 100% and 56.8% in the LT-LLH group and 100% and 54.8% in the GA-LLH group. The 1- and 3-year DFS rates were 93.3% and 61.3% in the LT-LLH group and 88.9% and 64.3% in the GA-LLH group. There were no significant differences between the groups regarding the 1- and 3-year OS rates or the 1- and 3-year DFS rates.

## Discussion

Laparoscopic left hepatectomy is one of the most common large-volume anatomical hepatectomies and is also a kind of mature laparoscopic hepatectomy. In recent years, many different laparoscopic left hemihepatectomy methods have been developed. From the early conventional extrahepatic hilar approach (15) to the extrahepatic Glissonean approach (16), and then to the recent Laennec's capsule approach (12), different hilar canal management methods are suitable for different cases. The hepatic parenchyma approach includes the hepatic parenchyma priority approach, the Arantius tube approach, the caudal ventral approach, the caudal dorsal approach, the dorsal cephalic approach (4, 17–19). Recently, Laennec's capsule approach has been widely used in liver surgery. Laennec's membrane can be used for peri-hepatic dissociation, hepatic pedicle separation, hepatic vein exposure, and anatomical hepatectomy (12, 20, 21). Sugioka et al. proposed that the Glisson pedicle of almost all liver segments can be separated to realize anatomical hepatectomy based on the anatomical concept of Laennec's capsule (11). Given of the development of various surgical methods, we combined Laennec's capsule Glissonean approach with the caudal-ventral liver parenchyma approach to explore a novel laparoscopic left hemihepatectomy called Laennec's membrane-tunnel laparoscopic left hepatectomy. Compared with the traditional extrahepatic Glissonean approach, shorter operation time and comparable long-term results were observed by performing the above procedure.

We summarized the key points of Laennec's membrane tunnel laparoscopic left hemihepatectomy as follows. First, blocking the first hepatic hilum with the Springle method can reduce bleeding and keep the operative field clear when separating the hepatic hilar plate. Second, accurate judgment of the location of Gate III is the premise of the successful operation. The position of Gate III is generally located at the bifurcation of the left and right hepatic pedicles, near the left edge of the right anterior Gleason sheath or the left edge of the right anterior bile duct. Third, before establishing the tunnel, it is necessary to find the gap between the Laennec's membrane and the liver tissue, that is, the Laennec's capsule. The liver tissue and blood vessels of the hepatic portal plate were separated by ultrasonic knife, and part of the liver parenchyma was split if necessary. Then the tissue was pushed and removed by a suction device, and the tunnel was established and enlarged. Forth, one end of the endoscopic cutter was inserted into the tunnel, the other end was placed in the ventral side of the Arantius ligament, and the left hepatic pedicle was severed with an extrahepatic Glissonean approach. To avoid damaging the middle hepatic vein, the head of the cutter should be moved to the left and upward direction. This procedure can be mainly used in patients with benign left liver tumors or malignant tumors who do not need to undergo hepatic duodenal ligament lymph node dissection and caudate lobectomy, as well as patients with left liver stones located a certain distance from the trunk of the left hepatic duct. This procedure provides a new surgical concept for laparoscopic left hemihepatectomy, which is only applicable to some patients. Intrathecal dissection can be considered when the Laennec's capsule cannot be effectively dissected due to excessive tumor size, local inflammation, and liver atrophy.

**Figure 4:**
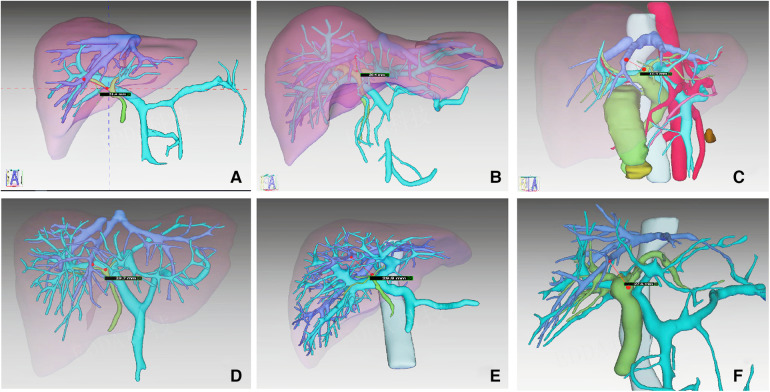
The distance between the entrance of the left hepatic duct and the main trunk of the middle hepatic vein in six cases were measured from the results of 3D.

We consider this technique to be safe. Through preoperative MRCP examination and three-dimensional reconstruction, we can accurately evaluate the shape of the biliary tract and blood vessels, so as to analyze whether there are anatomical variations, such as the opening of the right anterior lobe bile duct originating from the left hepatic duct (22). Before severing the left hepatic pedicle, we can clamp the left hepatic pedicle and observe the ischemic line to confirm whether it is the left hemihepatectomy line, combined with intraoperative ultrasound. The transection of the left hepatic pedicle is performed on the ventral side of the Arantius canal. Since the portal vein of the caudate lobe mostly comes from the transverse and angular parts of the left portal vein (23), the chance of severing these ducts is very small, and the liver pedicle of the caudate lobe can be well protected. Through preoperative three-dimensional reconstruction, we calculated that the average distance between the entrance to the liver of the left hepatic duct and the main trunk of the middle hepatic vein was more than 2.0 cm (Figure 4). The head of the endoscopic cutter was upward to the left, which could completely avoid damaging the middle hepatic vein. Compared with the traditional extrahepatic Glissonean approach, LT-LLH has obvious advantages in the total operation time, the time of left hepatic pedicle disconnection, and the time of hepatic parenchyma disconnection, which makes the operation faster. Moreover, only one side of the membrane tunnel needs to be established without completely separating the Glissonean sheath, and the left hepatic pedicle can be directly cut off with an endoscopic cutter, which makes the operation very convenient. Therefore, the main advantages of this operation are safe, faster, and convenient.

Although this study provides a new surgical procedure for laparoscopic left hemiliver, it also has its limitations. This study is a retrospective analysis with a small sample size, which may introduce potential selection bias. Although we introduce the PSM method to minimize the selection deviation, the mixed variables can not be completely avoided. Therefore, further multicenter prospective or retrospective large-sample studies and long-term follow-ups are needed to confirm these results.

## Conclusion

Compared with the traditional extrahepatic Glissonean approach, the LT-LLH approach is a safe, rapid and convenient operation procedure with shorter operation time and similar tumor prognosis to provide a novel surgery program for selected patients. However, this method is not suitable for all cases, and intrathecal dissection should be considered when liver atrophy, hilar displacement, inflammatory edema, and tumor compression make it impossible to separate the Laennec's capsule effectively.

## Data Availability

The original contributions presented in the study are included in the article/**Supplementary Material**, further inquiries can be directed to the corresponding author.
